# Interdisciplinary Management of Supracrestal Tissue Attachment Violation: A 5‐Year Follow‐Up Case Report

**DOI:** 10.1155/crid/6266425

**Published:** 2026-02-13

**Authors:** Elyan Al Machot, Ioannis Konstantinidis, Barbara Noack

**Affiliations:** ^1^ Policlinic of Operative Dentistry, Periodontology, and Pediatric Dentistry, Faculty of Medicine and University Hospital Carl Gustav Carus at TUD Dresden University of Technology, Dresden, Saxony, Germany; ^2^ Department of Prosthodontics, Faculty of Medicine and University Hospital Carl Gustav Carus at TUD Dresden University of Technology, Dresden, Saxony, Germany

**Keywords:** case report, gingival inflammation, periodontal tissue, preprosthetic periodontal plastic surgery, supracrestal tissue attachment

## Abstract

This clinical report describes an interdisciplinary approach adopted to treat esthetic and biological issues caused by violation of the supracrestal tissue attachment (SCTA). For the reestablishment of the SCTA and the improvement of the smile line, the surgical procedure was prosthetically driven and based on the desired outcome. The inappropriate crowns were replaced with provisional crowns until the maturation of the periodontal tissue was ensured. The final rehabilitation was completed with porcelain‐veneered zirconia single crowns. The 5‐year follow‐up demonstrated a stable esthetic and functional outcome.

## 1. Introduction

A fundamental requirement for the esthetic and functional rehabilitation of severely compromised teeth with crowns is the respect of the periodontium [1]. The violation of the supracrestal tissue attachment (SCTA), also known as biologic width, causes iatrogenic periodontal damage [2]. According to early studies by Gargiulo et al. [3], the SCTA is about 2.04 mm and includes the sum of the connective tissue and the epithelium that is attached to the tooth [4]. A systematic review on biologic width in restorative dentistry concluded that a universal dimension of biologic width does not exist. Variations in biologic width dimensions are influenced by factors such as tooth type, anatomical site, the presence of restorations, and the effects of periodontal diseases or surgical interventions [5]. For periodontal health, maintenance of the restorative margins within 0.5–1 mm in the gingival sulcus and approximately 3 mm coronal to the bone level as sufficient distance is pivotal for ensuring the health of the periodontium [6]. When the restoration margins are located too far below (0.5–1 mm) the gingival tissue, it will impinge on the gingival attachment and will facilitate constant inflammation in the area [7].

A recent comprehensive narrative review has extensively addressed the topic of biologic width and its clinical implications, emphasizing the need for updated strategies in restorative procedures. It highlights the importance of respecting biologic width in treatment planning and presents revised clinical guidelines based on current evidence [8].

In addition to mechanical damage to the periodontal tissue caused by the deeper placement of the restoration margins, the restoration acts as a plaque retention force due to the inability of the patient to clean an area deep below the gingival margin [9]. Furthermore, the mechanical and biological trauma affects the stability of the periodontal tissue [10]. In patients with thin periodontal phenotype [11], a common finding is the development of recessions due to the resorption of the marginal bone as an attempt to create space between the marginal bone and the restoration margins for the reestablishment of the SCTA [12]. However, in cases with a thick periodontal phenotype, the crestal bone may remain stable and a gingival inflammation develops and persists. In those cases, the gingiva shows permanent signs of inflammation such as erythema, swelling, and bleeding [13]. To reestablish a physiological SCTA for damaged teeth and achieve an effective ferrule effect, three options may be considered: crown lengthening, orthodontic extrusion, and surgical extrusion. Crown lengthening is regarded as an invasive procedure, while both orthodontic and surgical extrusion can help avoid this problem. The choice of therapy is determined by the specific advantages and disadvantages of each approach, as well as by the associated treatment time, contraindications, and potential complications [2, 14, 15]. In general, orthodontic extrusion is considered advantageous over surgical crown lengthening because it allows the achievement of a favorable crown‐to‐root ratio while preserving the alveolar bone support of adjacent teeth and maintaining the esthetic appearance of the involved tooth [16]. Compared with orthodontic or surgical extrusion, crown lengthening provides a more direct and time‐efficient approach, while avoiding prolonged treatment phases or risks such as root resorption and ankylosis. These considerations supported the choice of crown lengthening in this case, ensuring a biologically sound and clinically practical outcome.

The SCTA exhibits considerable interpatient and intertooth variability [17]. Considering the case‐ and tooth‐specific characterization, it is recommended to assess the SCTA through bone sounding. These measurements should be carefully considered when performing a surgical crown lengthening procedure. This approach allows clinicians to reestablish the SCTA according to the new position of the restoration margins [5].

This case report describes an interdisciplinary approach to treat a patient who suffered from the violation of the SCTA caused by inappropriate crowns.

## 2. Case Presentation

**Figure 1:**
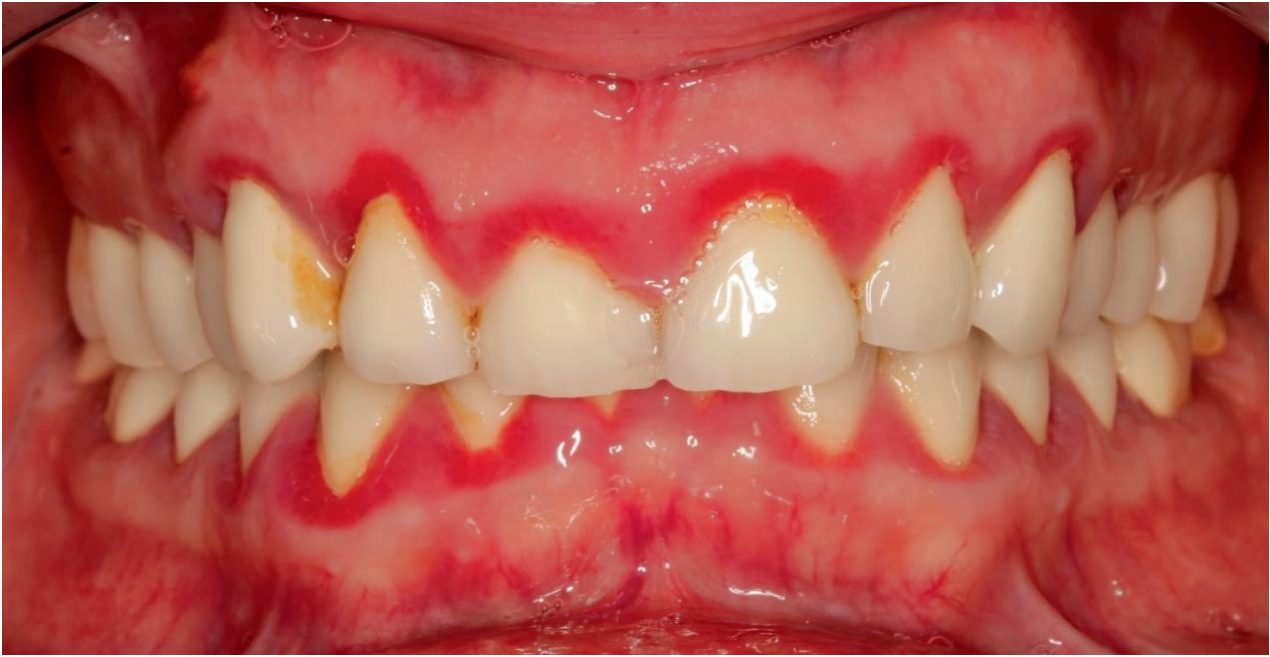
Initial clinical situation.

**Figure 2:**
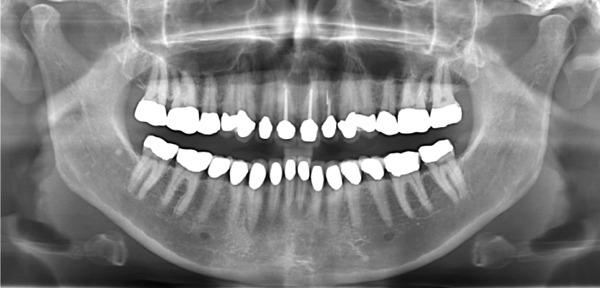
Initial radiographic examination.

A 40‐year‐old female patient presented to the Division of Periodontology at the University hospital Carl Gustav Carus at Technical University Dresden TUD, Germany, due to dissatisfaction with the esthetic appearance of her smile. In particular, she was primarily concerned about the asymmetrical gum line and the overall appearance of her gums, which were markedly red and swollen on a permanent basis. The patient presented with no significant medical history; however, she has suffered from bruxism since an early age, associated with periods of psychological stress that had led to excessive tooth wear and chipping of previous restorations. At the time of presentation, she was not wearing an occlusal splint. Additionally, she stated that 10 years prior to seeking treatment, all her teeth had been restored with crowns. Furthermore, it was reported that several months after the placement of the crowns, the patient presented with gingival redness and swelling, with evidence of bleeding. Despite the regular periodontal care that her dentist provided, she did not see any improvement. The clinical examination revealed a thick periodontal phenotype and the presence of severe gingivitis localized in the maxillary and mandibular teeth, while the posterior teeth exhibited stability and no evidence of bleeding on probing (Figure 1). Based on the clinical signs, it was suspected that the main cause of the periodontal problem was the violation of the SCTA. Accordingly, a bone sounding with periodontal probe (PCP 15, Hu‐Friedy Mfg. Co. LLC, Chicago, Illinois, United States) was performed under local anesthesia. The examination showed that the crowns on the maxillary and mandibular incisors, the maxillary premolars, and maxillary left lateral incisor had an inappropriate marginal adaptation. In the marginal area of these teeth, a gap between the crown margins and the tooth was apparent. Additionally, the distance between the crown margin and the crestal bone of the affected teeth was, on average, 1.5 mm, and the margins of crowns in the maxillary and mandibular front teeth exhibited overcontouring. Moreover, the crown on the maxillary right molar showed signs of chipping. Radiographic examination showed the presence of periapical periodontitis on Teeth 13, 31, 43, 45, and 46 as well as insufficient root canal treatment on Teeth 11, 21, and 22 (Figure 2).

The aim of treatment was to reestablish the SCTA, to treat the endodontically affected teeth, and to renew restorations on the maxillary and mandibular front teeth and premolars in order to provide stable periodontal tissue and a new symmetrical gummy line that meets the patient′s esthetic requirements.

The treatment plan involved a multidisciplinary approach and included dental hygiene, root canal treatment of the affected teeth, gingival level adjustment by surgical crown lengthening on most teeth and coronally advanced flap on tooth 23, subsequently preparation and fabrication of porcelain veneered zirconium oxide single crowns for the maxillary and mandibular premolars and anterior teeth. The treatment timeline was carefully developed to ensure proper coordination of surgical, restorative, and prosthetic phases, taking into account the healing time of periodontal tissues to achieve stable, long‐term results without complications. Following the diagnosis and formulation of the treatment plan, the patient was duly informed and provided written consent for the execution of the treatment.

**Figure 3:**
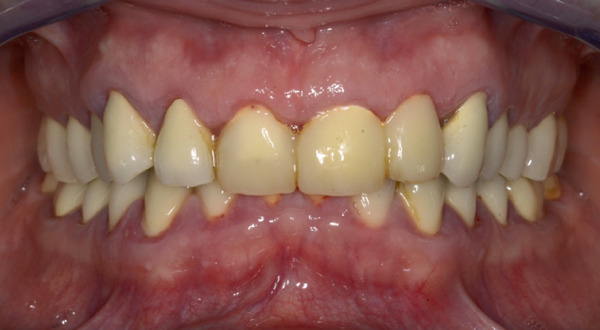
Clinical situation with provisional crowns of Teeth 11, 21, and 22 after professional dental cleaning and oral hygiene instructions.

Following a professional mechanical plaque removal and detailed instruction in oral plaque control as the basic foundation for further successful treatment, the crowns of Teeth 11, 21, 22 that violated the SCTA were removed and replaced with provisional crowns. Provisional crowns were fabricated conventionally using an alginate impression taken immediately before the endodontic treatment and were cemented chairside after completion of the procedure. The margins of the provisional crowns were located 3 mm above the marginal bone. Next, root canal treatments of Teeth 13, 11, 21, 22, 31, 43, 45, and 46 were performed by an endodontist. The patient was scheduled for a follow‐up appointment approximately 3 weeks after the placement of provisional crowns. At this appointment, there was a notable improvement in the periodontal tissue situation (Figure 3).

**Figure 4:**
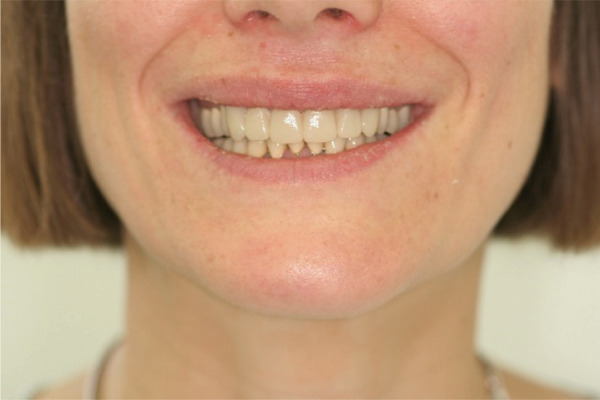
Mock‐up.

Following the completion of the root canal treatments, the diagnostic wax‐up using a plaster model was assembled. The initial diagnostic wax‐up was additive, with the objective of creating a mock‐up over the existing restorations and ensuring that the patient′s esthetic requirements were met in terms of smile line symmetry and tooth proportions. Following confirmation from the patient that she was satisfied with the mock‐up (Figure 4), the surgical crown lengthening procedure was initiated. A surgical guide was fabricated using a transparent thermoformed splint based on the wax‐up model with the objective of achieving a symmetric gummy line and controlling the surgical crown lengthening process. The splint can be easily adjusted to the individual anatomy of the patient [18].

The SCTA was measured on all teeth through bone sounding using a periodontal probe PCP 15 directly before starting the surgical procedure. Subsequently, the surgical guide was positioned on the teeth to direct the incision. Paramarginal and sulcular incisions were performed both at the buccal and lingual site of the teeth, and the gingival tissue was removed. A flap was prepared between 16 mesial and 26 mesial in the maxillary jaw and in the mandibular jaw from distal 45 to distal 35. The flap was of full thickness coronally and split thickness apically. Following the elevation of the flap, it became evident that the crowns exhibited insufficient marginal adaptation and that the SCTA was violated in certain areas. Using special KaVo SonicFlex tips (KaVo Dental Excellence, Germany), osteotomy and osteoplastic around the affected teeth were performed and then root planning was executed for the prevention of the reattachment (Figure 5). Finally, external vertical mattress sutures were placed to stabilize the periodontal tissue apically. On the maxillary left canine, a coronally advanced flap was stabilized with a sling suture with the aim to have a symmetrical gummy line compared with Tooth 13. The patient was instructed to rinse twice daily with 0.12% chlorhexidine digluconate solution. Following a 14‐day period, the patient returned for removal of sutures (Figure 6).

Figure 5Surgical crown lengthening in the (a) maxillary and (b, c) mandibular jaw highlighting the exposure and correction of the tissues involved.

**Figure:**
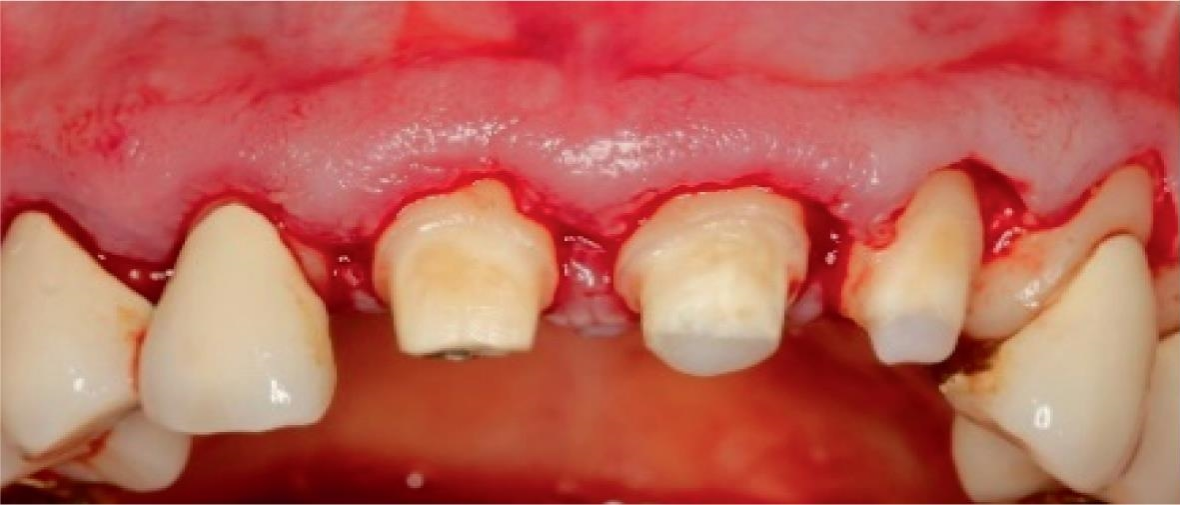
(a)

**Figure:**
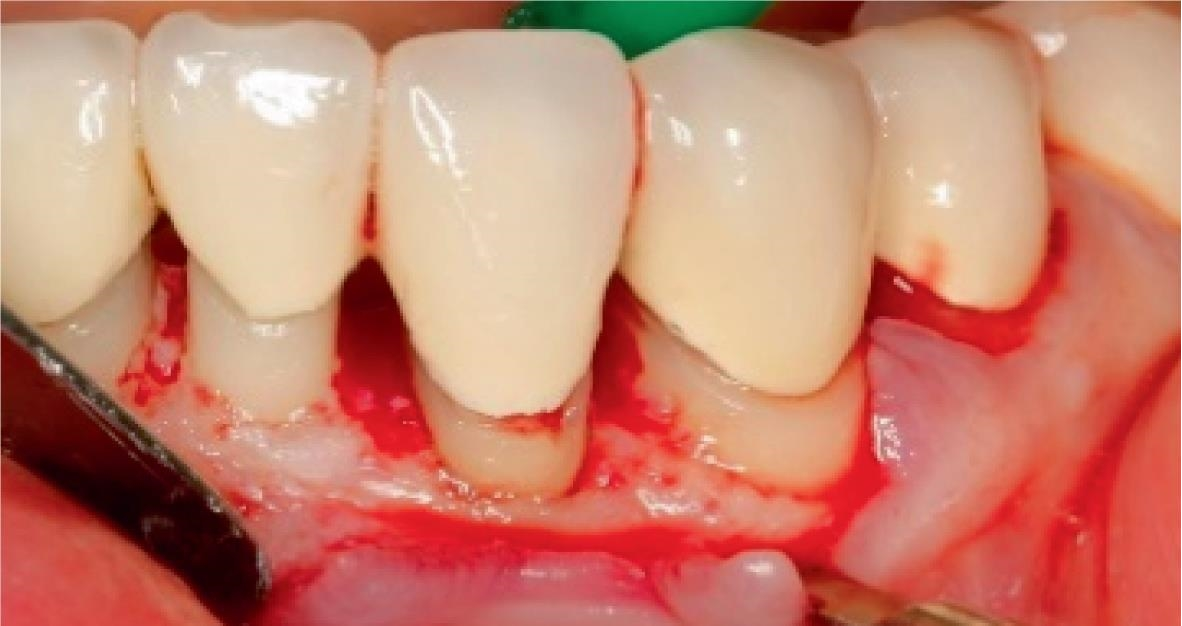
(b)

**Figure:**
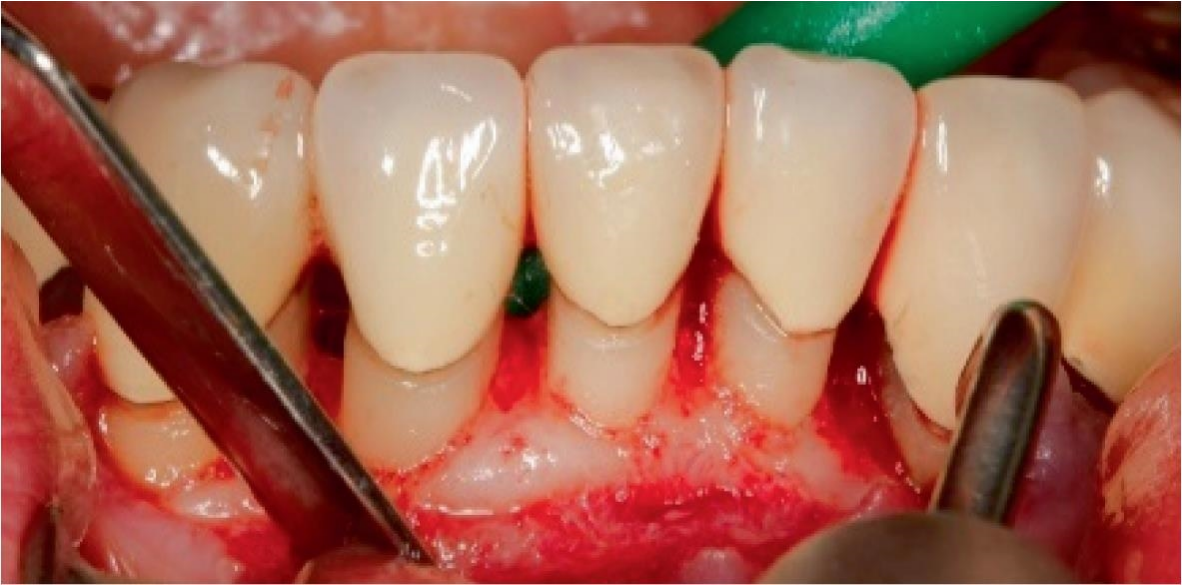
(c)

Figure 6Clinical situation 14 days after surgical procedure in the (a–c) maxillary and (d–f) mandibular aspect.

**Figure:**
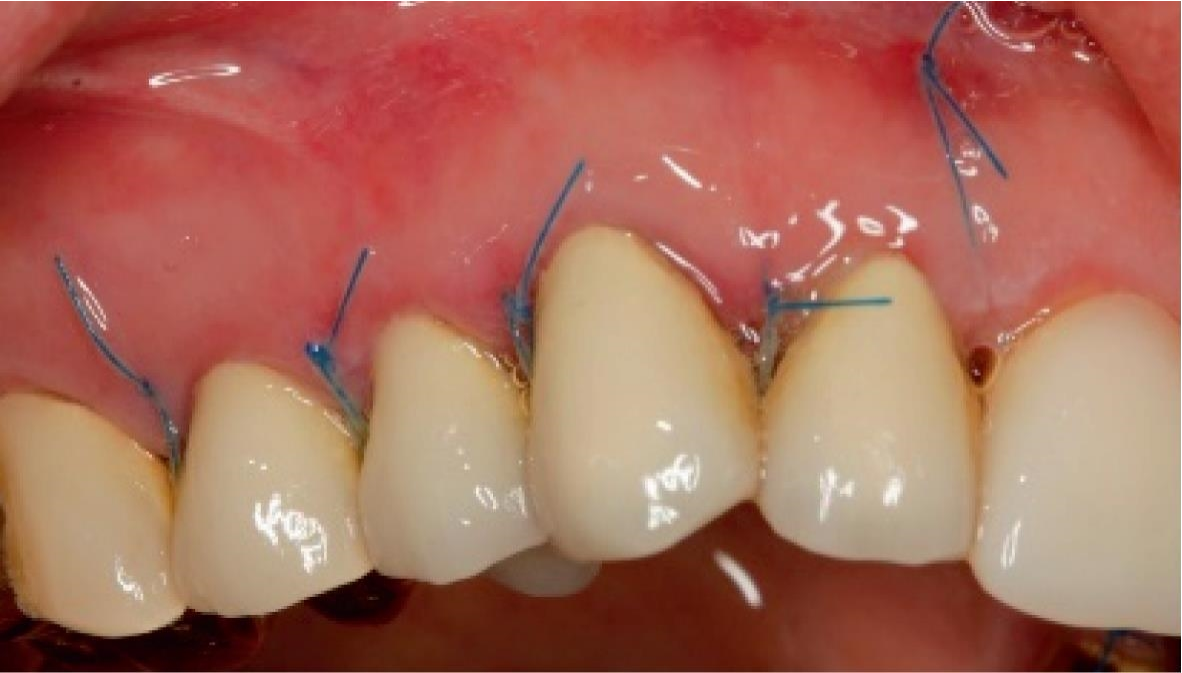
(a)

**Figure:**
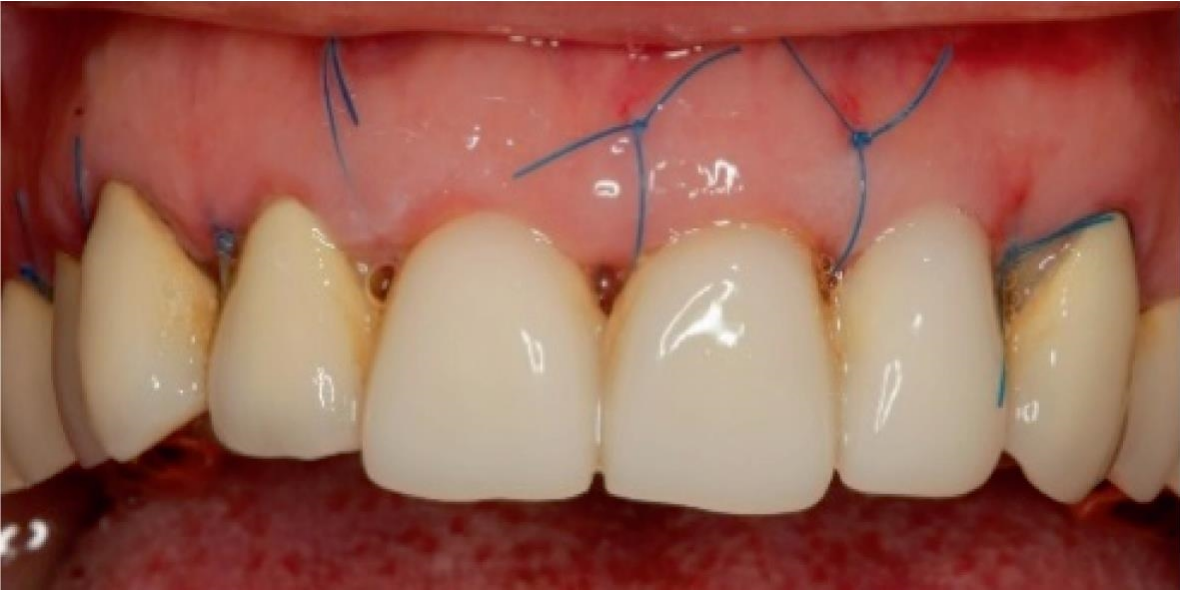
(b)

**Figure:**
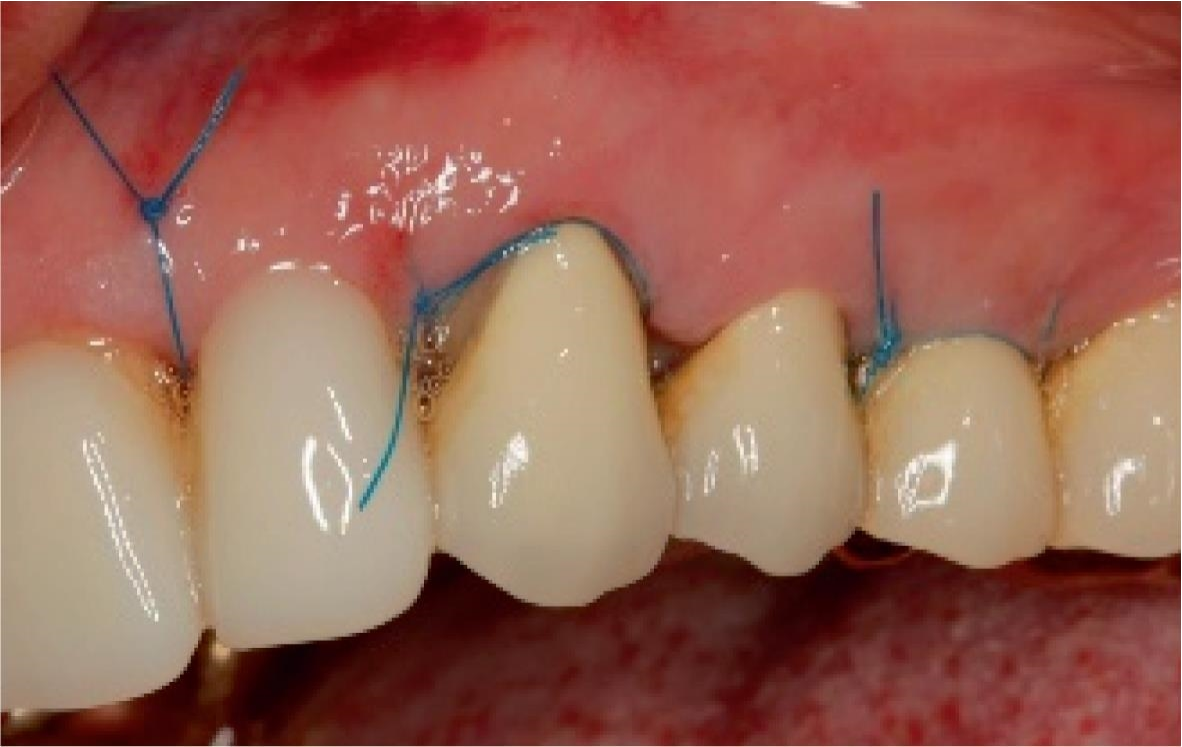
(c)

**Figure:**
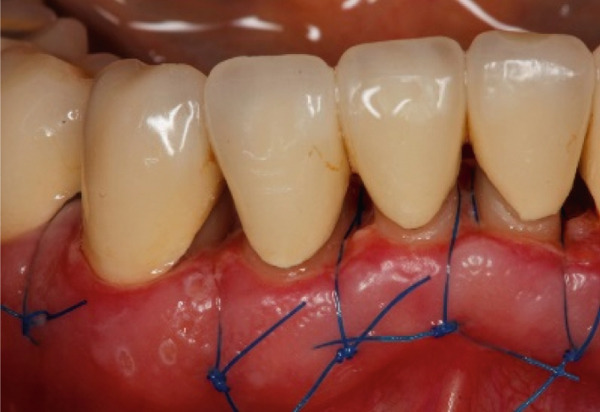
(d)

**Figure:**
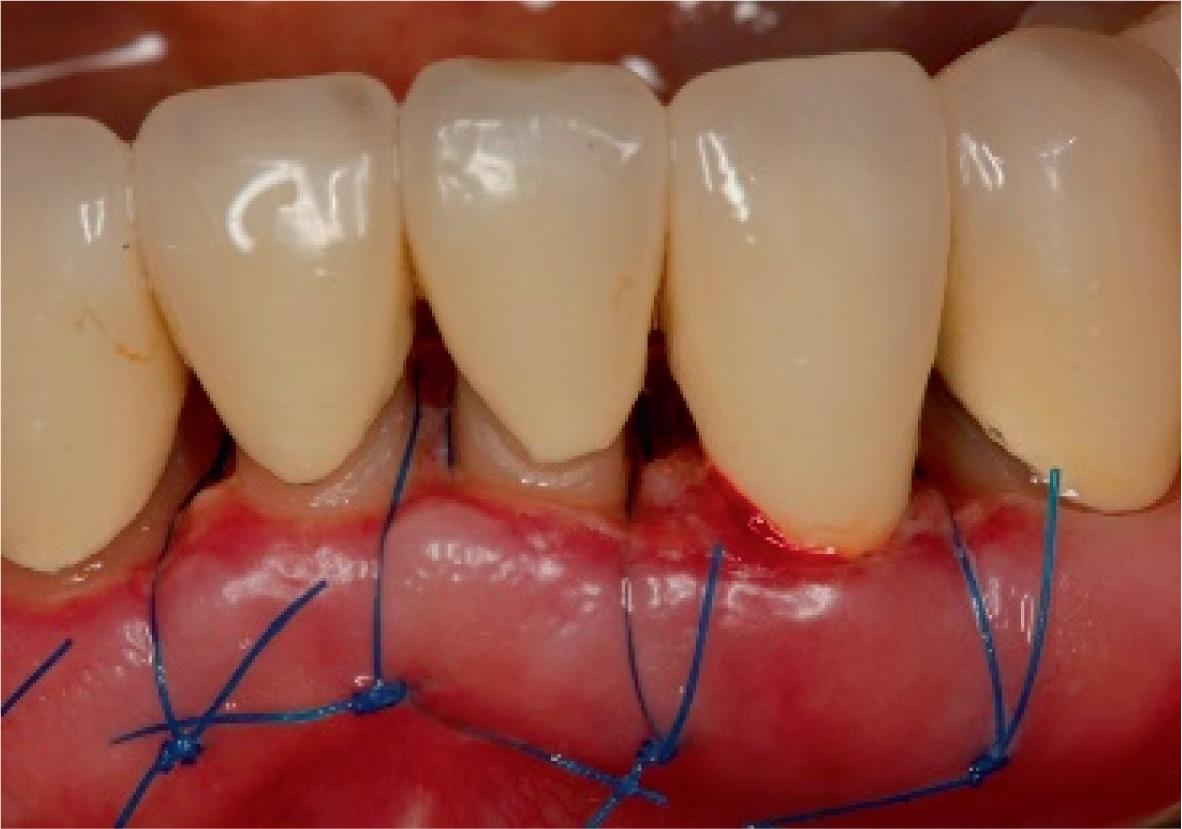
(e)

**Figure:**
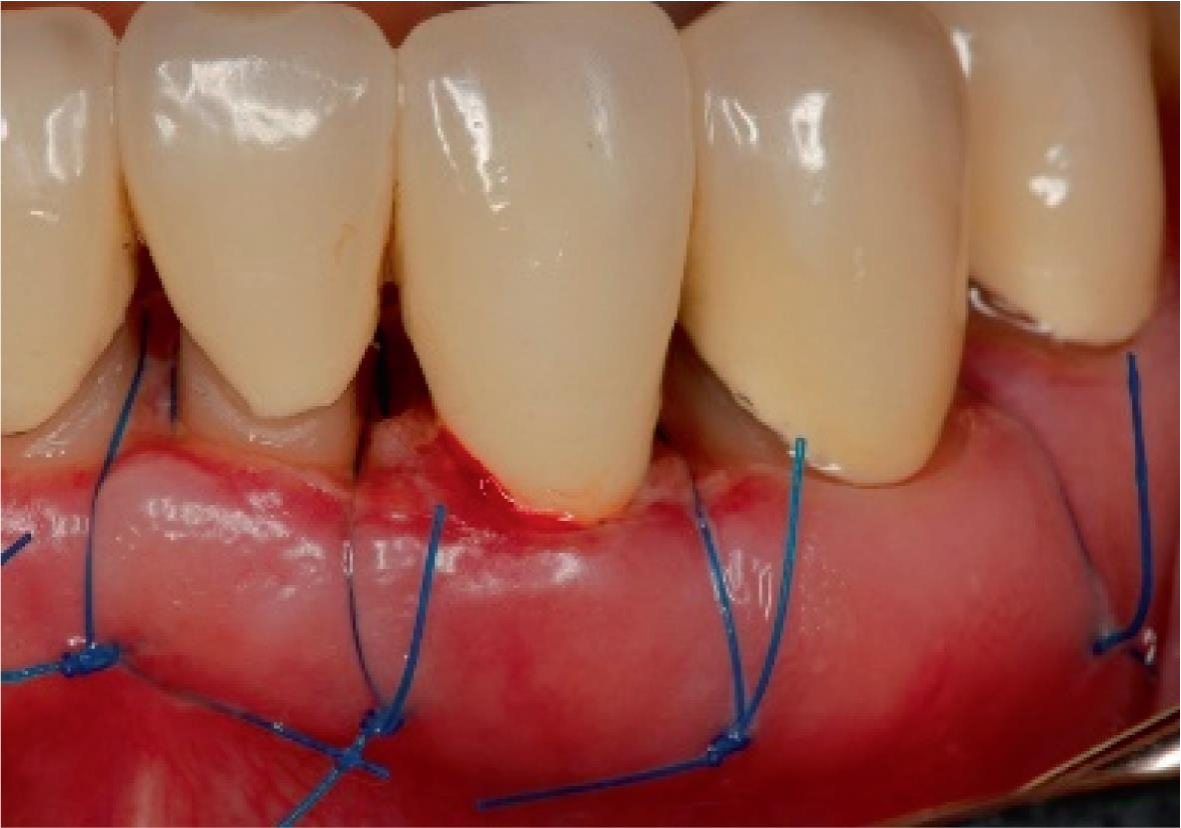
(f)

Three months after the surgical procedure, the old crowns were removed and the initial provisional crowns from the maxillary and mandibular front teeth and premolars as well as the crown from the first maxillary right molar were installed. Utilizing a matrix from the initial diagnostic wax‐up, provisional crowns were fabricated directly with bis‐acrylic resin.

Provisional crowns were maintained for 2 months, during which occlusion was monitored biweekly. This period was obligatory for the complete maturation of the periodontal tissue and for the confirmation that the patient tolerated the form of the provisional crowns.

**Figure 7:**
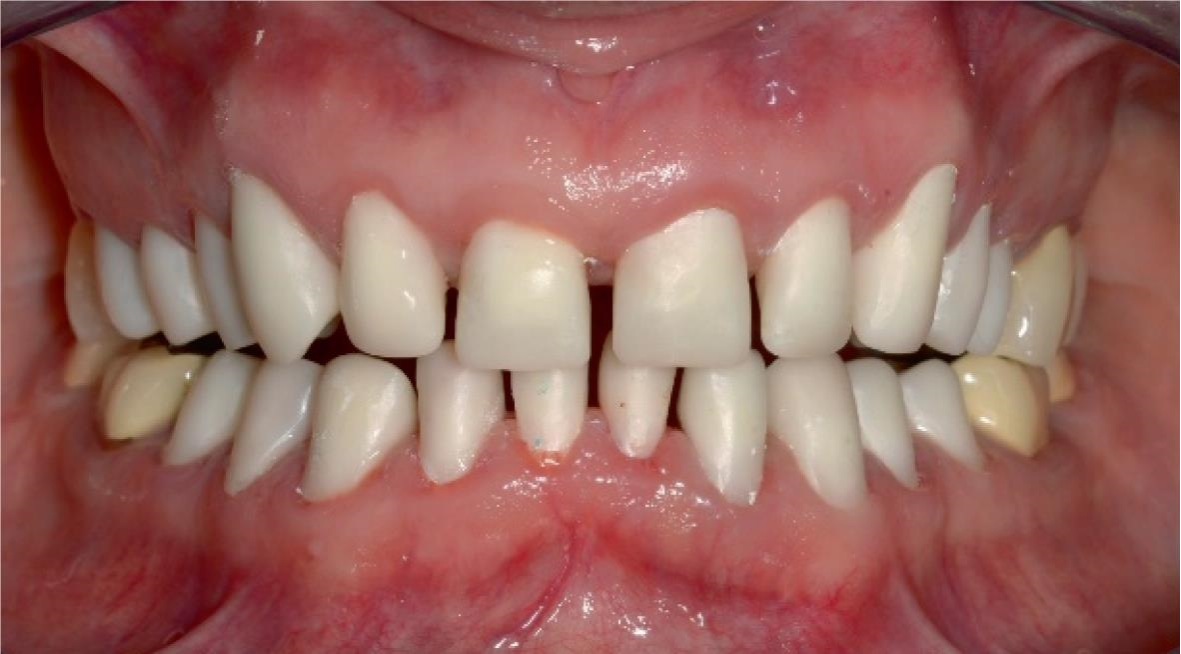
Fit control of the zirconia frameworks.

**Figure 8:**
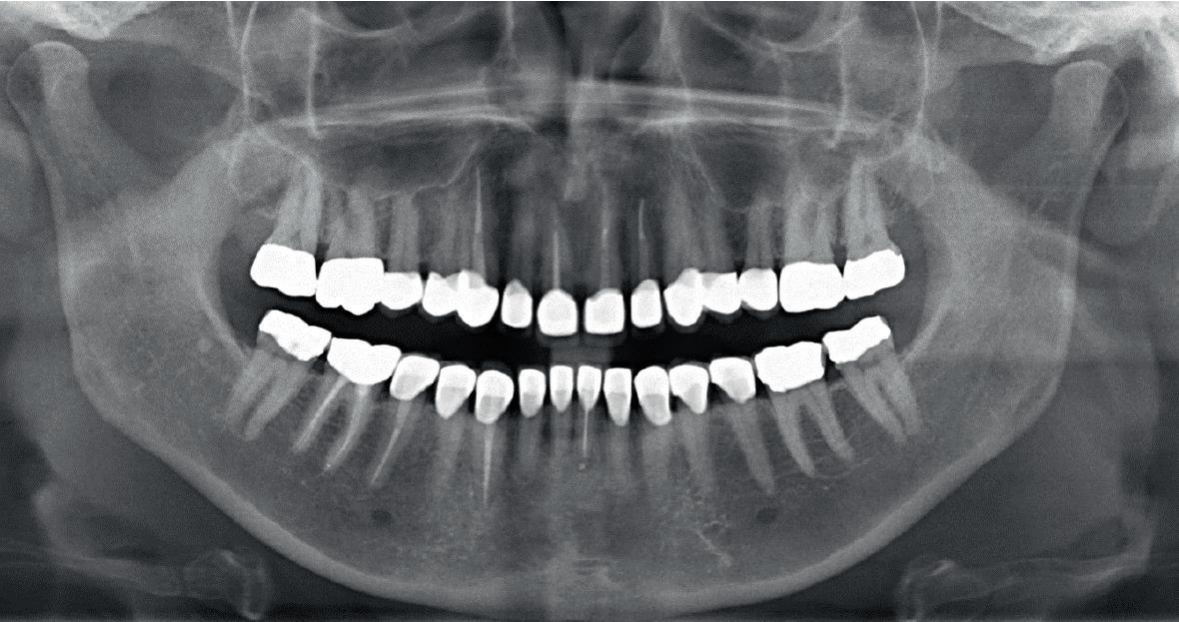
A panoramic x‐ray 5 years after treatment.

Once the final preparation of the teeth had been completed, impressions with A‐silicon were taken using the double cord technique. To transfer the contours and occlusion from the provisionals to the final restorations, the cross‐mounting technique was used. The model of the prepared teeth was mounted on an adjustable articulator and it was scanned in the laboratory. Zirconia‐veneered restorations were selected for this case due to their high fracture resistance and long‐term wear resistance, making them advantageous over lithium disilicate in high‐load applications. Compared to monolithic zirconia, they offer superior esthetics, including natural translucency, customizable color, and reduced abrasiveness to opposing teeth [19, 20]. Combining strength and esthetics, zirconia‐veneered restorations are a versatile choice, particularly for this case with bruxism, endodontically treated teeth, and high esthetic demands [21]. The zirconia frameworks of the single crowns were fabricated with CAD/CAM technique (Figure 7). The frameworks were checked for the adaptation and then they were veneered with porcelain. The occlusion was designed to provide canine guidance, and the crowns were adhesively cemented using Panavia V5 (Kuraray Noritake Dental Inc., Tokyo, Japan). An occlusal splint was fabricated after completion of the definitive rehabilitation and prescribed as a preventive measure against bruxism‐related complications. At follow‐up, the patient did not report further bruxism activity, and the splint was primarily intended to ensure the long‐term stability of the restorations. The patient was incorporated into a recall protocol on a twice‐yearly basis to maintain motivation and adherence, develop skills in removing supragingival dental biofilm, and perform professional mechanical plaque removal along with risk factor control. Plaque index by O′Leary et al. [22] was recorded and consistently remained below 25%. After 5 years of follow‐up, there was an improvement in the radiographic findings following endodontic and periprosthetic treatment (Figure 8), with no clinical evidence of gingivitis or any technical or other biological complications. The surgical technique used resulted in recession coverage at Tooth 23 and a symmetrical gingival height in the anterior teeth in both the maxillary and mandibular jaws. The clinical periodontal parameters indicated a stable condition, with pocket probing depths of ≤ 3 mm in all teeth, bleeding on probing below 10%, and no attachment loss compared to the results obtained 6 months after prosthetic treatment. In comparison to baseline, the selection of appropriate interdisciplinary procedures resulted in improvements in both periodontal findings and esthetics including harmonious soft tissue architecture with a harmonious gingival line (Figures 9 and 10).

Figure 9Porcelain fused to zirconia cemented on teeth, clinical situation 5 years after treatment. (a) Vestibular aspect of both arches, (b) approximated right aspect, and (c) approximated left aspect.

**Figure:**
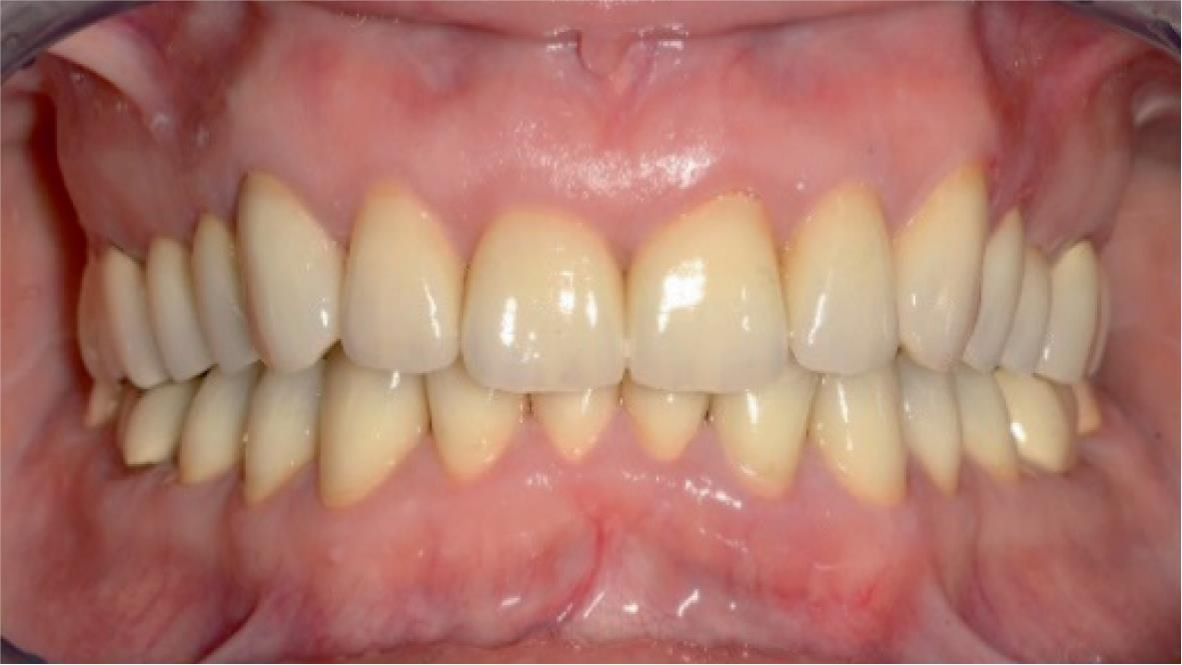
(a)

**Figure:**
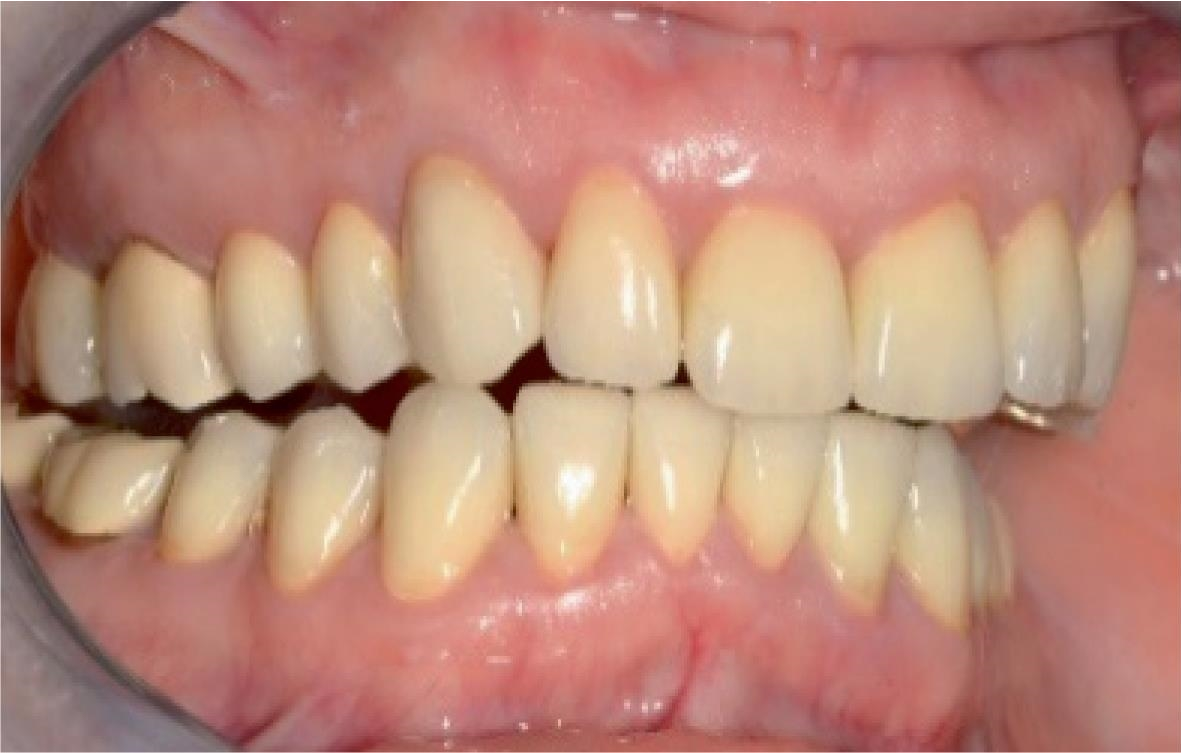
(b)

**Figure:**
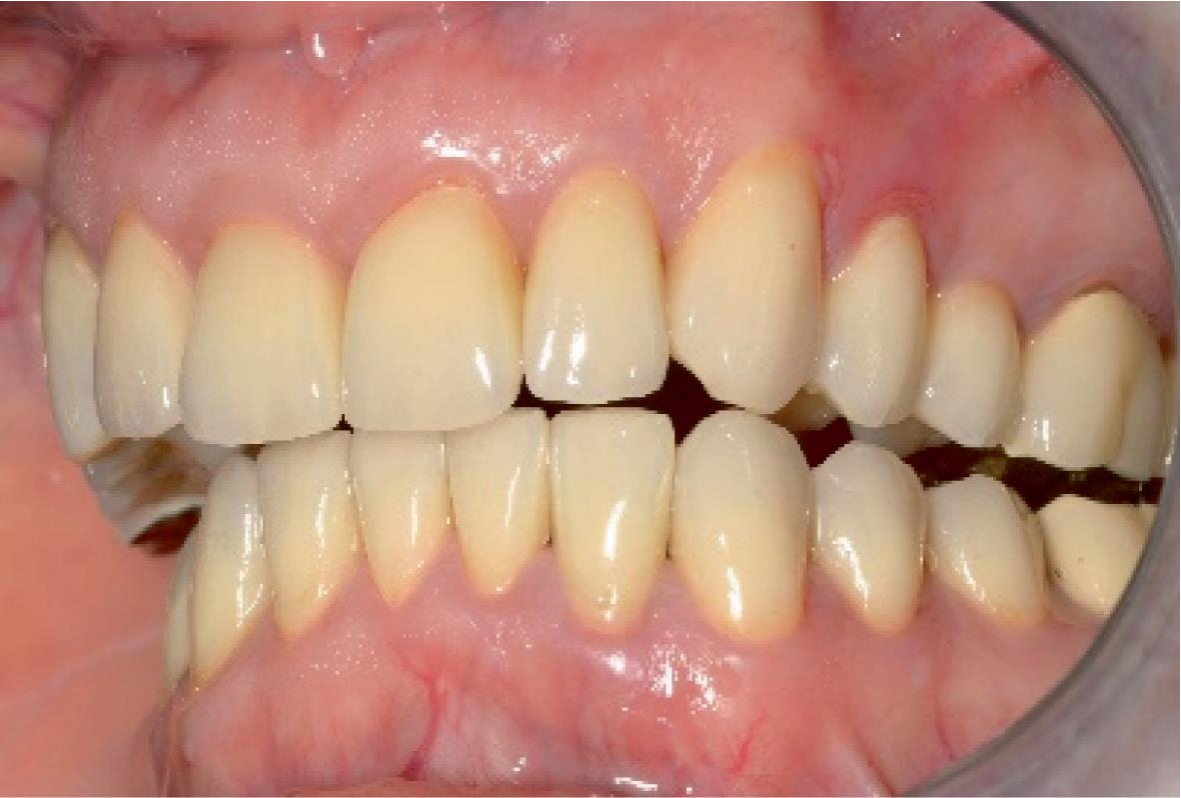
(c)

Figure 10Extraoral photographs of the smile. (a) Front aspect (b) approximated right aspect and (c) approximated left aspect.

**Figure:**
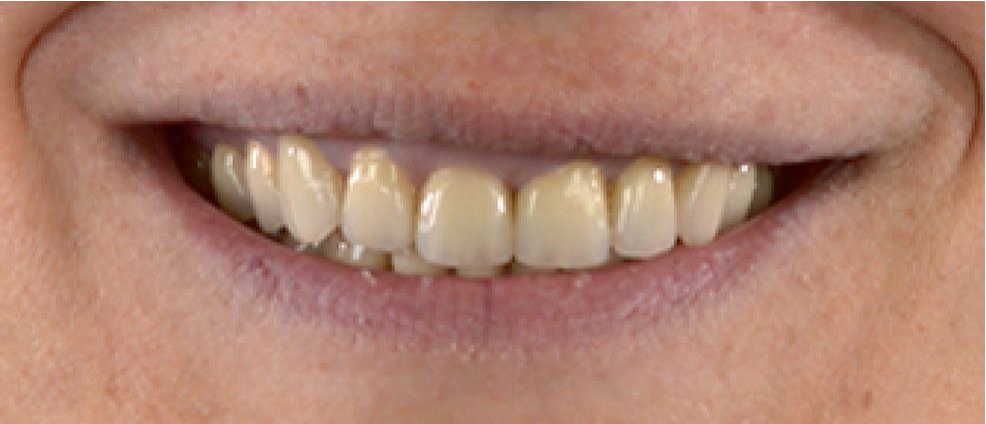
(a)

**Figure:**
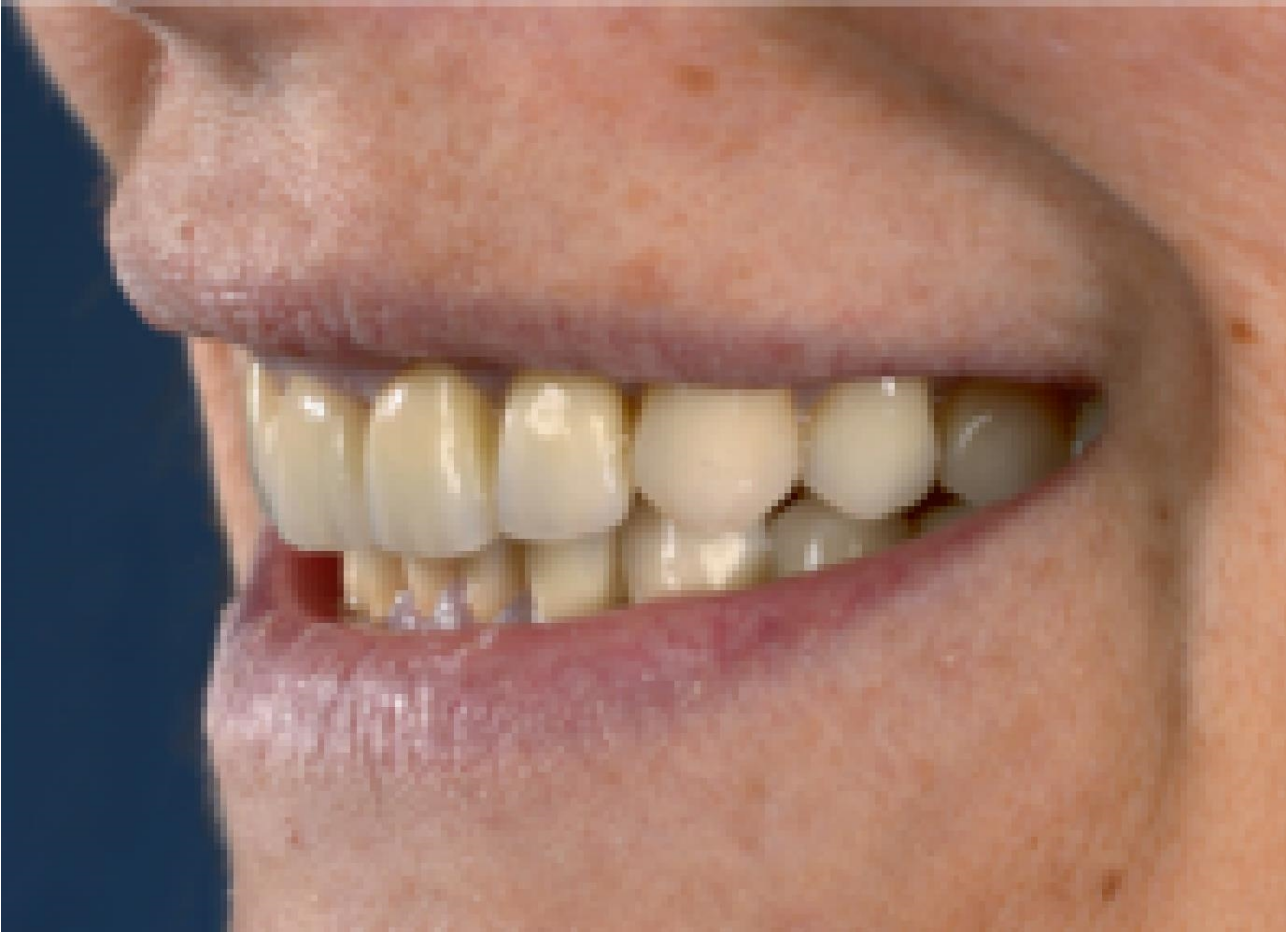
(b)

**Figure:**
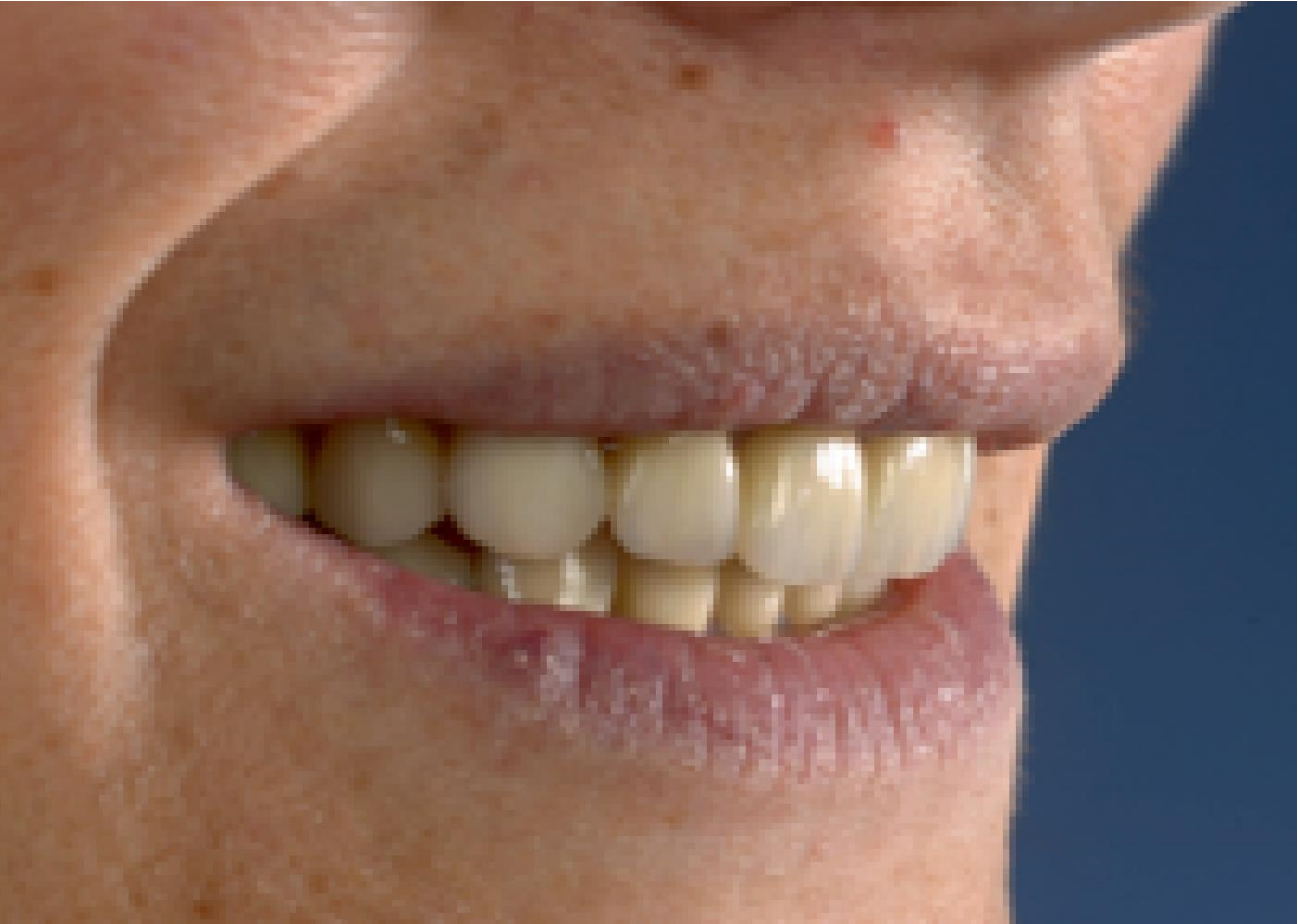
(c)

## 3. Discussion

This case report presents a multidisciplinary treatment approach to achieve a biological, functional, and esthetic outcome. The patient presented herself for treatment due to concerns regarding the appearance of her gums. This was determined to be a result of a violation of the SCTA. In cases where the SCTA in the anterior teeth has been violated, particularly when there is an esthetic component, a widely supported clinical approach is traction or root extrusion. This provides an excellent outcome in prosthetically rehabilitated teeth [23]. In view of the extensive nature of the clinical situation, which involved numerous teeth and extended beyond the esthetic area, as well as the necessity for an extensive rehabilitation regimen, this approach was not deemed appropriate. In this case, the teeth were prepared too deeply subgingivally to achieve the necessary retention for the crowns and to create teeth with the correct proportions. The deep subgingival tooth preparation complicates the impression process, which may have contributed to the inappropriate marginal adaptation of the crowns [24]. However, we decided to perform a crown lengthening procedure because we could correct the asymmetrical gummy smile that was bothering the patient. According to the patient‐reported outcomes, the patient treated in this case was satisfied with the results of the procedure and tolerated the surgical interventions well [25]. An important tool for this successful surgical process was the surgical guide fabricated from the diagnostic wax‐up. Numerous surgical guide designs have been described in the literature [26–28]; however, stability and access for incision are key requirements.

According to established principles of wound healing and supporting literature, tissue maturation and healing typically occur within 8–12 weeks when a buccal flap is elevated and bone is exposed. In cases involving bone removal, a minimum of 6 months is required for adequate soft tissue stabilization. In the present case, a 6‐month healing period was observed to allow for the maturation of the periodontal tissues, ensuring a stable and predictable outcome [29]. During this period, the teeth were restored with provisional crowns. The material of choice for the prosthetic rehabilitation of the patient was zirconia veneered with laminate porcelain. This material shows excellent mechanical and biological stability [30, 31]. However, the stability of the final result is largely dependent on the patient′s compliance and oral hygiene that need to be established at the beginning of treatment [32]. The 5‐year follow‐up showed stable results due to the patient′s adherence to treatment. The restorative design significantly influences tissue response and esthetic integration.

## 4. Conclusion

This case demonstrates that SCTA damage resulting from incorrectly positioned restoration margins can be successfully corrected through carefully planned preprosthetic periodontal plastic surgery along with integrating well‐designed and located crown margins. Patient education regarding the importance of meticulous plaque control for periodontal maintenance is essential. It is of the utmost importance to develop a tailored, patient‐specific treatment plan that considers both periodontal and prosthetic factors, as this will enhance the outcome and patient satisfaction, leading to better long‐term dental health.

## Author Contributions

Conceptualization: Elyan Al Machot. Methodology: Elyan Al Machot and Ioannis Konstantinidis. Investigation: Elyan Al Machot and Ioannis Konstantinidis. Data curation: Elyan Al Machot. Writing—original draft: Elyan Al Machot and Ioannis Konstantinidis. Writing—review and editing: Elyan Al Machot and Barbara Noack. Resources: Elyan Al Machot. Visualization: Elyan Al Machot. Project administration: Elyan Al Machot.

## Funding

No additional funding was received. The described treatment occurred within the scope of the employment relationship with the University Hospital Carl Gustav Carus, Dresden, Germany, where all authors were employed during the treatment. Open Access funding enabled and organized by Projekt DEAL.

## Ethics Statement

The patient involved in this case report provided written consent to the procedures and the publication of the material.

## Conflicts of Interest

The authors declare no conflicts of interest.

## Data Availability

The data that support the findings of this study is available on request from the corresponding author. The data is not publicly available due to privacy or ethical restrictions.
